# Possible Benefit of Angiotensin II Receptor Blockers in COVID-19 Patients: A Case Series

**DOI:** 10.1155/2021/9951540

**Published:** 2021-05-13

**Authors:** Su Jin Lee, Taehwa Kim, Woo Hyun Cho, Doosoo Jeon, Seungjin Lim

**Affiliations:** ^1^Division of Infectious Disease, Department of Internal Medicine, Pusan National University Yangsan Hospital, Yangsan, Republic of Korea; ^2^Research Institute for Convergence of Biomedical Science and Technology, Pusan National University Yangsan Hospital, Yangsan, Republic of Korea; ^3^Division of Pulmonology, Allergy and Critical Care Medicine, Department of Internal Medicine, Pusan National University Yangsan Hospital, Yangsan, Republic of Korea

## Abstract

**Introduction:**

Dysfunction in the renin-angiotensin-aldosterone system (RAAS) has been observed in patients with coronavirus disease 2019 (COVID-19). It is presumed that the effect of reducing interleukin-6 (IL-6) levels by angiotensin II receptor blockers (ARBs) by RAAS modulation. We investigated changes in angiotensin II and IL-6 levels in four COVID-19 patients treated with ARBs. *Case Presentation*. Cases 1 and 2 were who had not received ARBs before and were newly administered ARBs. Case 3 restarted ARBs after discontinuation for 7 days, and case 4 received an increased dose of ARBs. The mean in angiotensin II levels (607.5 pg/mL, range: 488–850 pg/mL, reference range < 100 pg/mL), C-reactive protein (CRP) (10.58 mg/dL, range 4.45-18.05 mg/dL), and IL-6 (55.78 pg/mL, range: 12.86–144.82 pg/mL, reference range < 7 pg/mL) was observed at the admission in all patients. Upon clinical improvement, the mean decrease in CRP (1.02 mg/dL, range 0.06-3.78 mg/dL) and IL-6 (5.63 pg/mL, range 0.17-20.87 pg/mL) was observed in all patients. Conversely, angiotensin II levels gradually increased.

**Conclusion:**

This report supports the potential benefit of ARBs to improve the clinical outcomes of COVID-19 patients by controlling RAAS dysfunction.

## 1. Introduction

Severe acute respiratory syndrome coronavirus 2 (SARS-CoV-2) binds to target cells through the angiotensin-converting enzyme 2 (ACE2), which is expressed by the epithelial cells of the lungs, intestine, kidney, and blood vessels [1, 2]. Further, patients with hypertension have increased ACE2 activity and are treated with ACE inhibitors (ACEi) and angiotensin II receptor blockers (ARBs) [1]. Although some experts suggest that ACE2-stimulating drugs increase the risk of severe coronavirus disease 2019 (COVID-19) [1], others recommend that patients should continue antihypertensive therapy because there is no clinical evidence to suggest that treatment with ACEi or ARBs should be discontinued in cases of COVID-19 infection [2]. Moreover, previous studies propose that ARBs and ACEi may help attenuate lung injury caused by a cytokine storm [3, 4]. Angiotensin II induces oxidative stress and the expression of inflammatory cytokines, such as interleukin-6 (IL-6) [5]. ACE2 is a key counterregulatory enzyme that degrades angiotensin II to angiotensin-(1–7), thereby attenuating the effects of angiotensin II on vasoconstriction, sodium retention, and fibrosis [6]. Therefore, it is presumed that ARBs can reduce tissue injury. Notably, it was also observed that severe COVID-19 patients had increased levels of angiotensin II and IL-6 [7, 8]. This can be potentially explained by the fact that the ACE2 that is occupied and downregulated by SARS-CoV-2 is incapable of hydrolyzing angiotensin II [9].

Based on previous studies [3, 4, 6, 7], we hypothesized that patients with COVID-19 pneumonia may have high levels of angiotensin II, because SARS CoV-2 causes ACE2 dysfunction in the human body [10], and that ARBs could improve the prognosis of COVID-19 by regulating the renin-angiotensin-aldosterone system (RAAS) [3]. This study is aimed at investigating the effect of ARBs in patients with severe COVID-19. We measured the angiotensin II and IL-6 levels of COVID-19 patients who were administered ARBs. Furthermore, we evaluated the correlation between changes in IL-6 and C-reactive protein (CRP) levels and clinical improvement.

Our hospital is a tertiary hospital that treats patients with severe COVID-19. The plasma and serum were collected from patients according to a predetermined schedule (days 1–3, days 4–9, days 10–13, and days 14–20 of hospitalization). The measurement of plasma angiotensin II (Phoenix Pharmaceutical; Burlingame, CA, USA) and serum IL-6 (R&D Systems Inc; Minneapolis, MN, USA) levels was performed after discharging patients. The clinical characteristics and treatment of patients are shown in Table 1, and the initial laboratory findings are shown in Table 2. This study was reviewed and approved by the Institutional Review Board of Pusan National University Yangsan Hospital (05-2020-068) and performed in accordance with the ethical standards laid down in the 1964 Declaration of Helsinki and its later amendments. Informed consent was obtained from all patients prior to inclusion in this study.

## 2. Case Presentation

### 2.1. Case 1

**Figure 1:**
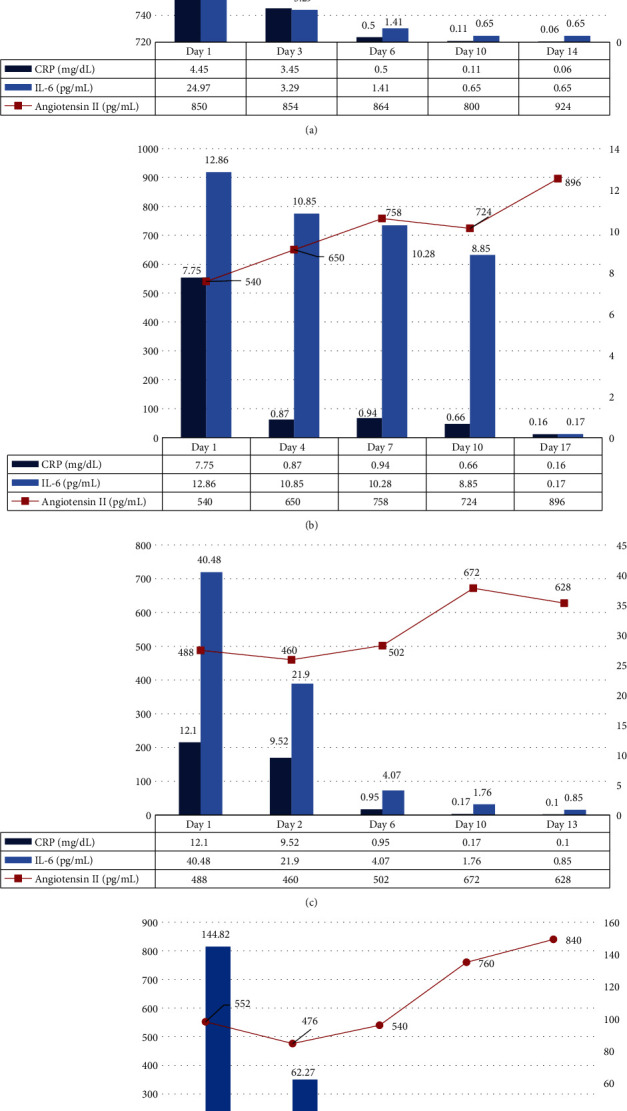
Angiotensin II, C-reactive protein, and interleukin-6 levels over time in case 1 (a), case 2 (b), case 3 (c), and case 4 (d).

A 28-year-old man was transferred to our hospital due to the worsening of COVID-19 pneumonia after 12 days of illness. He had no underlying comorbidity. His initial vital signs were as follows: blood pressure (BP) 138/76 mmHg, heart rate (HR) 81 bpm, respiration rate (RR) 22 breaths/min, body temperature (BT) 38.5°C, and oxygen saturation (SpO_2_) 89%. He had dyspnea on exertion. After supplying 3 L of oxygen, the patient's SpO_2_ was 96%. We administered losartan (50 mg/day), levofloxacin, and lopinavir/ritonavir. Two days after admission, lopinavir/ritonavir was changed to hydroxychloroquine (400 mg/day) because of diarrhea. His symptoms improved 3 days after hospitalization. Oxygen supplementation was stopped 7 days after hospitalization. On day 15 of admission, he was discharged without complication. Changes in angiotensin II and IL-6 levels were investigated by blood specimens collected from day 1 of admission until discharge. The angiotensin II levels increased from 850 pg/mL at admission to 924 pg/mL at discharge. In contrast, CRP and IL-6 levels decreased progressively until discharge (Figure 1(a)).

### 2.2. Case 2

A 71-year-old man with unmedicated hypertension and diabetes was admitted for the management of COVID-19 pneumonia. Despite the administration of lopinavir-ritonavir during prior hospitalization, pneumonic infiltration had progressed as observed on a chest X-ray performed after 11 days of illness. Besides high blood pressure (175/86 mmHg) and low SpO_2_ (94%, 3 L oxygen), other vital signs were stable (HR 86 bpm, RR 20 breaths/min, BT 36.4°C). Hydroxychloroquine and losartan (100 mg/day) were administered on day 1 of hospitalization. The patient's condition gradually improved, and the CRP level normalized on day 4. He was discharged after 34 days. His angiotensin II and IL-6 levels before increasing the ARB dosage were 540 pg/mL and 12.86 pg/mL, respectively. Angiotensin II levels gradually increased to 896 pg/mL at 17 days after admission. IL-6 levels changed from 12.86 pg/mL to 0.17 pg/mL, and CRP levels also decreased from 7.75 mg/dL to 0.16 mg/dL at 17 days after admission (Figure 1(b)).

### 2.3. Case 3

A 49-year-old man with hypertension was admitted for management of COVID-19 pneumonia after 11 days of illness. His initial body temperature was 39.5°C, and SpO_2_ was 87% on room air. Other vital signs were as follows: BP 111/75 mmHg, HR 80 bpm, and RR 20 breaths/min. With inhalation of 5 L of oxygen, SpO_2_ increased to 94%. He had dyspnea, cough, and fever. He took telmisartan and amlodipine (40 mg/day and 2.5 mg/day, respectively) for 3 years: however, these medications were discontinued for 7 days after admission. He was administered losartan (50 mg/day) and hydroxychloroquine. On day 3 of admission, the losartan dosage was increased to 100 mg/day. The patient no longer had a fever 3 days after hospitalization and no longer needed oxygen therapy after 6 days of hospitalization. Testing for angiotensin II and IL-6 levels was performed before the redosing of ARBs. Initially, angiotensin II and IL-6 levels were 488 pg/mL and 40.48 pg/mL, respectively (Figure 1(c)). The IL-6 level decreased from 40.48 pg/mL to 21.9 pg/mL after two days of admission. Angiotensin II level increased gradually from 488 pg/mL to 628 pg/mL after 13 days of admission. In contrast, IL-6 and CRP levels decreased.

### 2.4. Case 4

A 78-year-old man with diabetes, hypertension, and ischemic heart disease was transferred to our hospital due to worsening COVID-19 pneumonia after 12 days of illness. He had fever, dyspnea, cough, and myalgia. Initial vital signs on admission were as follows: BP 158/94 mmHg, HR 109 bpm, RR 20 breaths/min, BT 38.7°C, and SpO_2_ 88% on room air. Although already on ARBs (50 mg/day losartan, 5 mg/day amlodipine, and 12.5 mg/day thiazide), systolic blood pressure was above 150 mmHg. Losartan dosage was increased to 100 mg on day 3 of hospitalization. His fever and dyspnea gradually improved. He was discharged 27 days after hospitalization. IL-6 and angiotensin II levels were measured before changing the dosage of losartan (angiotensin II; 552 pg/mL, IL-6; 144.82 pg/mL). IL-6 levels decreased to 24.5 pg/mL at 9 days after admission. Angiotensin II levels increased from 552 pg/mL to 840 pg/mL at 16 days after admission. In contrast, IL-6 levels decreased from 12.86 pg/mL to 0.17 pg/mL, and CRP levels decreased from 18.05 mg/dL to 3.78 mg/dL (Figure 1(d)).

## 3. Discussion

This study reported four COVID-19 patients admitted to our hospital. After obtaining informed consent, first blood samples were taken before ARBs administration at the time of admission. Finally, we evaluated changes in angiotensin II, IL-6, and CRP levels in four patients who were administered ARBs to determine the change of angiotensin II due to ARBs in COVID-19 patients. Cases 1 and 2 were newly administered ARBs, while case 3 was restarted on ARBs. Case 4 was already on ARBs, and the dosage was increased to control his blood pressure. All patients had a reduced SpO_2_ of below 90% on room air before being transferred to our hospital. The mean angiotensin II and IL-6 levels were higher than the reference range (angiotensin II: 607.5 pg/mL, range 488–850 pg/mL, reference range < 100 pg/mL, and IL-6: 55.78 pg/mL, range 12.86–144.82 pg/mL, reference range < 7 pg/mL) in all the cases. During hospitalization, the patients' conditions consistently improved with decreasing IL-6 and CRP levels. The high levels of IL-6 observed in this study are consistent with those reported in severe COVID-19 patients in a previous study [8]. In contrast, angiotensin II levels were observed to increase gradually in all patients during hospitalization until discharge.

A recent study suggests that ACE2 is occupied and downregulated by SARS-CoV-2 and is, therefore, incapable of hydrolyzing angiotensin II [9]. The attachment of SARS-CoV-2 to ACE2 is positively correlated with angiotensin II levels [9]. Angiotensin II induces inflammatory cytokine expression and markers, such as IL-6 and CRP in humans [11]. IL-6, stimulated by angiotensin II, leads to increased NADP and NADPH production, altering vascular permeability, constriction, and fibrosis degree [12]. These findings suggest that ACEi and ARBs may have a protective role against angiotensin II-mediated organ damage during COVID-19 infection [11].

The effect of ARBs on IL-6 level reduction has been proposed in previous studies on patients with hypertension or diabetes and other conditions [13–16]. Angiotensin II induces oxidative stress, activates nuclear factor *κ*B (NF-*κ*B), and induces the expression of inflammatory cytokines and markers such as IL-6 and high-sensitivity C-reactive protein (hsCRP) [5]. Angiotensin II level is high in patients using ARBs, but it cannot show its effect because it cannot bind to its receptor [17]. Angiotensin II type 1 receptor antagonism reduces serum concentration of the inflammatory markers IL-6 and hsCRP [5]. Based on findings from previous studies and our report, we suggest that ACEi or ARBs function by controlling RAAS and the modulation of the levels of cytokines, such as IL-6 in patients with COVID-19. Since high levels of IL-6 have been associated with lung lesions in SARS-CoV-2 infection [18], ARBs could have potential benefits of reducing IL-6 levels.

Our study has some limitations. First, it is a case series consisting of only four patients. Second, the reduction in IL-6 level might not have occurred due to ARBs alone but also due to the administration of hydroxychloroquine [19]. However, this is the first report showing the serial measurements of angiotensin II and IL-6 levels during the treatment of COVID-19 pneumonia patients. High levels of angiotensin II and IL-6 were observed in all four patients. We suggest that ARBs have a potential benefit in preventing organ damage in patients with COVID-19.

Since consistently high angiotensin II was observed after SARS-CoV-2 infection in severe cases, the duration of administration of ARBs should be discussed in the future to prevent end-organ injury. In addition, we propose the need for further studies on this subject. First, studies are needed to investigate changes in angiotensin II and IL-6 levels in patients with ARBs compared to that in patients not administered ARBs even after advances in treatment with remdesivir and dexamethasone. Second, because outpatient follow-up was not possible in our center during the early stages of COVID-19 pandemic, the patient's chronic symptoms after COVID-19 could not be confirmed. It will also be meaningful to study the correlation between the presence of chronic COVID-19 symptoms and persistent angiotensin II increase after discharge.

## Figures and Tables

**Table 1:** Clinical characteristics and treatments of patients.

	Patient			
	Case 1	Case 2	Case 3	Case 4
Sex	Male	Male	Male	Male
Age (y)	28	71	49	78
Weight (kg)	85	75	83	69
Incubation period	Unknown	Unknown	9	Unknown
Interval between symptomonset and admission (days)	12	11	15	12
Initial symptoms	Fever, myalgia	Fever, myalgia, cough, sputum	Fever, myalgia	Fever, myalgia, cough, sputum
Symptoms at admission	Fever, dyspnea, myalgia, cough, sputum	Fever, dyspnea, myalgia, cough, sputum	Fever, dyspnea, myalgia, cough, sputum	Fever, dyspnea, myalgia, cough, sputum, chest discomfort, diarrhea, headache
Smoking	None	Ex-smoker	None	None
Underlying disease				
Diabetes	None	Yes	None	Yes
Hypertension	None	Yes	Yes	Yes
Ischemic heart disease	None	None	None	Yes
Chest X-ray	Focal consolidation in both lower lobes	Diffuse patchy consolidation	Diffuse patchy consolidation	Diffuse patchy consolidation
Oxygen demand (L)	3	3	5	3
Treatment				
Antiviral agent				
Lopinavir/ritonavir	Yes	Yes	Yes	Yes
Hydroxychloroquine	Yes	Yes	Yes	Yes
Antibiotics				
Piperacillin/tazobactam	No	Yes	Yes	Yes
Levofloxacin	No	Yes	Yes	Yes
Azithromycin	Yes	No	No	No

**Table 2:** Initial laboratory findings of patients.

	Patient			
	Case 1	Case 2	Case 3	Case 4
Laboratory findings on admission				
WBC (/mm^3^)	2890	5260	3980	4580
Neutrophil (%)	66.1	50.7	83	85.8
Lymphocyte (%)	22.5	35.2	12.3	7.6
Hemoglobin (g/dL)	14.6	11.6	18.9	12.5
Platelets (10^3^/mm^3^)	113	159	113	153
AST (IU/L)	28	28	32	55
ALT (IU/L)	18	20	23	39
LDH (U/L)	294	275	283	249
BUN (mg/dL)	13.7	9.7	7.9	16.1
Cr (mg/dL)	0.89	0.77	0.96	0.87
CRP (mg/dL)	4.45	7.75	12.1	21.31
Procalcitonin (ng/mL)	0.065	<0.06	<0.06	0.226
Ct value of COVID-19 RT-PCR^∗^	36.33	33.97	24.76	29.65

WBC: white blood cells; AST: aspartate aminotransferase; ALT: alanine aminotransferase; LDH: lactate dehydrogenase; BUN: blood urea nitrogen; Cr: creatinine; CRP: C-reactive protein; Ct: cycle threshold; RT-PCR: reverse transcription-polymerase chain reaction.

## Data Availability

All data generated or analyzed during this study are included in this published article.
